# Humanistic Optimal Functioning Predicts Low Youth Violence

**DOI:** 10.1177/00221678221132331

**Published:** 2022-11-16

**Authors:** Roger G. Tweed, Gira Bhatt, Stephen Dooley, Jodi L. Viljoen, Kevin S. Douglas, Nathalie Gagnon

**Affiliations:** 1 Kwantlen Polytechnic University, Surrey, BC, Canada; 2 Simon Fraser University, Surrey, BC, Canada; 3 Simon Fraser University, Burnaby, BC, Canada

**Keywords:** humanistic, violence, aggression, positive regard, at risk, authenticity, gratitude

## Abstract

This study assessed whether indicators of humanistic optimal functioning were predictive of lower levels of violence among youth across a 6-month period. Youth (*N* = 346) aged 12 to 14 years completed measures of authenticity and of positive regard for others (generalized trust, forgiveness, and gratitude). Approximately 6 months later, the youth reported violence, criminal offenses, and indicators of potential violence, and for some (*n* = 266), a teacher provided ratings of aggression. Authentic living, some elements of generalized trust, forgiveness, and gratitude predicted lower levels on indicators of aggression or violence or readiness for violence 6 months later. The relation between humanistic predictors and violence-related outcomes was larger for youth at elevated risk for violence. Unexpectedly, a subtype of authenticity, “resisting external influence,” predicted higher violence, but other outcomes were in the expected direction. Thus, a humanistic lens may have value in examinations of societal violence.

This longitudinal study assessed whether indicators of humanistic optimal functioning were predictive of lower levels of violence-related outcomes among youth across a 6-month period. To humanistic psychologists, it may seem self-evident that humanistic ideals, such as authenticity, positive regard for others, and other indicators of optimal development will reduce violence because psychological health will negate the need for violence. Consistent with this view, humanistic approaches to violence have been proposed (e.g., Atieno Fisher, 2003).

Although the impact of humanistic optimal functioning on violence may seem self-evident for some humanistic psychologists, the ideas seldom receive mention from violence researchers (Coupland & Olver, 2020). If the relevance of humanistic psychology was broadly evident to people not identifying as humanistic psychologists, then one would expect constructs, such as authenticity and positive regard, to be central in both violence theory and assessment.

The relative dearth of attention is understandable, given that, to outsiders, attention to violence may appear contrary to humanistic psychology’s essence. Maslow (1954), for example, is often noted for his famous claim that research focused on stunted, immature, and unhealthy participants will produce a disabled psychology. However, humanistic psychologists may know that in the book containing that famous quote, Maslow also wrote extensively about psychotherapy, indicating his great interest in and concern for pathology. Similarly, Carl Rogers (2012) engaged extensively with therapeutic work, again indicating attention to pathology. He also addressed racism, overpopulation, over-policing, and unjust war (Rogers, 1972), and others have followed this humanistic tradition of attention to pathology (Franco et al., 2020; Morrill, 2021).

Thus, there is value in generating data explicitly testing the link between humanistic ideals and violence. This study assesses predictive power across 6 months.

Violence researchers may be more open to humanistic concepts than they were in the past, as demonstrated by recent research on protective factors (Coupland & Olver, 2020; de Vogel et al., 2011; Muir et al., 2020). However, one must be careful when reading literature on protective factors because “protective,” as used within these sources, does not necessarily mean factors that cause reduced violence, but instead means factors predicting lower levels of violence. Protective factors include intelligence, positive attitude toward authority, leisure activities, positive home, school, and community environments, prosocial involvement, and social support (de Vogel et al., 2011; Muir et al., 2020; Zych et al., 2019). Clearly, positive environments are relevant to humanistic psychology, but given the humanistic focus on the optimal functioning within individuals, further attention to optimal functioning and protection may also deserve attention. Humanistic psychology includes attention to many variables, but only a few variables that seemed particularly relevant to violence were examined in this assessment.

Authenticity has long been a central construct for humanistic psychology (Sohmer, 2020). Wood et al. (2008) defined authenticity as “to know yourself and act accordingly.” This definition maps closely onto Rogers’ (1957) concept of congruence. Prior to conducting this study, we gathered a community coalition to provide feedback. They expressed concern about gang violence, so we included questions about gangs. They also suggested measuring authenticity. The theoretical link between authenticity and violence might not seem obvious; however, Rogers (1957) believed that incongruent people (i.e., people not acting congruently with their true self) are prone to anxiety and disorganization. If he was correct, then inauthenticity could predict lashing out in frustration from a disorganized personal state rather than living in a mindful state (Tohme & Joseph, 2020). In contrast, authenticity may promote tolerance of others who disagree (Sartre, 1975). In addition, authenticity involves a willingness to stand for one’s own beliefs (Wood et al., 2008), which may help youth resist peer pressure supporting violence.

Another central construct in humanistic psychology is positive regard for others. Carl Rogers (1980) believed that the mature person would emanate positive regard for others, and that it would positively affect others. He described potatoes that grew green stalks when planted in nutritious soil but when stored in the dark basement of his childhood home, they grew twisted white stalks. He argued that his clients were the same. If he, as an optimally functioning human, provided conditions of growth, including positive regard, the clients’ growth tendency could be trusted. Readiness to have positive regard for others underlies much of humanistic psychology.

Positive regard could possibly manifest as generalized trust, a belief that most people are trustworthy. Regions with higher belief that people are trustworthy have far lower rates of violence (Elgar & Aitken, 2011), possibly because low trust produces readiness to protect oneself against the anticipated malicious acts of others and, possibly, even readiness to use violence (Buber, 1957). In contrast, youth who have more positive views of human nature seem more likely to support world peace (Adams, 1989).

Generalized trust does not represent all of positive regard for others, so it is important to assess other indicators of positive regard (Tweed et al., 2021). For example, forgiveness involves a willingness to let go of resentment toward others who have wronged the self and, in contrast, hold a positive or at least not negative view of them. This too may protect against violence (García-Vázquez et al., 2020; Tweed et al., 2011), possibly because forgiveness displaces vengeful attitudes (Berry et al., 2005) that drive much violence (Kennedy, 2011).

Another indicator of positive regard is gratitude, a willingness to look in the past and recognize that another has given something of value to the self. It is a willingness to see positive aspects in another’s contribution to the self. Gratitude may have an equally important, but less obvious relation to violence (García-Vázquez et al., 2020; Tweed et al., 2011). Gratitude promotes prosocial intentions (Tsang, 2006) and predicts lower levels of antisocial behavior (Bono et al., 2019). Violent intentions may tend to dissipate if one experiences gratitude.

Indicators of optimal functioning may not elicit much interest from violence researchers unless they provide predictive power beyond that provided by widely used indicators of risk, so we also assessed risk in this study. Prior analyses indicated relevant predictive risk factors of violence, including school failure, impulsivity, and peer delinquency (Otto & Douglas, 2010).

The hypotheses for this study were as follows:

**Hypothesis 1 (H1):** Humanistic optimal functioning, as assessed with measures of authenticity and positive regard for others (generalized trust, forgiveness, and gratitude), predicts lower levels of violence and potential for violence 6 months later (partial correlations controlling for gender and age).
**Hypothesis 2 (H2):** Humanistic optimal functioning contributes longitudinally predictive power beyond that provided by the risk indicators [impulsivity, peer delinquency, and school failure] (tested with hierarchical linear regressions entering demographics, then risk factors, and then optimal functioning).
**Hypothesis 3 (H3):** The effect is comparable at varying levels of risk. In other words, in longitudinal prediction of violence-related outcomes, the interaction between optimal functioning and risk does not approach significance (i.e., *p* > .20).

## Method

### Participants

The 346 participants were aged between 12 and 14 years (*M* = 13.1, *SD* = .40) and included 157 males and 189 females. Participants were recruited from Grades 8 and 9 of public school classes in Western Canada. On average, 58% of the students received the required active parental consent and participated in the questionnaire. The most reported ethnicities were South Asian (38% of the sample), European (13%, although a further 4% reported their ethnicity as Canadian, and, based on the local demographics, many of them may have had European ethnicity), East Asian (5%, although a further 14% described themselves as Asian Canadian and we suspect many of these were East Asian), and Indigenous (4%). In addition, 14% did not report their ethnicity.

### Measures

#### Four Predictive Measures Were Collected at Time 1

##### Authentic Living and Authentically Resisting Influence (Wood et al., 2008)

This model of authenticity includes three constructs: being true to oneself (“authentic living”), resisting external influences, and not feeling alienated from oneself, and has some evidence for validity (Wood et al., 2008). Authentic living (four items, for example, “I think it is better to be yourself than to be popular,” α = .69) adheres closest to the authenticity construct that our community coalition suggested for the study. The second construct involves resisting influence from others (four items, for example, “I usually do what other people tell me to do,” reverse scored, so higher scores indicated authenticity; α = .77). For each item, possible responses ranged from 0 “not at all true” to 3 “very much true.” The self-alienation subscale was not included out of concern that the concepts would be too difficult for the youth of this age and could be conflated with dysphoria (e.g., “I feel alienated from myself”).

##### Generalized Trust

Trust was assessed with the trust items from the Monitoring the Future Study and the General Social Survey, which ask the participants whether most people “can be trusted,” “would try to be helpful,” or “would try to be fair.” Each item had three response options that varied by question (e.g., for the first item: “Can’t be too careful,” “Don’t know,” or “Most people can be trusted”). The item scores are typically combined to create one score that functions in theoretically meaningful ways and thus has evidence for validity (Brehm & Rahn, 1997; Elgar & Aitken, 2011; Rahn & Transue, 1998; Stavrova & Ehlebracht, 2016; Twenge et al., 2014). The items have had limited internal consistency reliability (α = .45) but have nonetheless shown some similar and expected patterns of relations with other variables (Oishi et al., 2011). In this study, however, because of the limited internal consistency, the trust items were not combined but were instead used as separate variables, as has been previously done by Oishi et al. (2011).

##### Trait Forgiveness Scale (Berry et al., 2005; Berry & Worthington Jr, 2001)

The Trait Forgiveness scale has 10 items, including “I can usually forgive and forget an insult” and “I feel bitter about many of my relationships.” Cronbach’s alpha was .72 in this sample. For each item, possible responses ranged from 0 “not at all true” to 3 “very much true.” Validity is supported by negative relations with vengeful rumination, and positive relations with third-party ratings of respondents and scenario-based measures of forgiveness (Berry et al., 2005; Berry & Worthington, 2001).

##### Gratitude Questionnaire-8 (GQ-8)

The GQ-8 is a youth version of the well-validated GQ-6 (McCullough et al., 2002) provided by an author of the GQ-6 (R. A. Emmons, personal communication, 2009). The GQ-6 has structural and convergent validity (Froh et al., 2011) with youth except for one item reflecting an extended time period (“Long amounts of time can go by . . .”). The GQ-8 replaces two items of this type with four new items (e.g., “When good things happen to me, I think of the people who helped me.”). In addition, the wording was slightly simplified by using “thankful” rather than “grateful.” Cronbach’s alpha was .77 in this sample. For each item, possible responses ranged from 0 “not at all true” to 3 “very much true.”

#### Five Outcome Measures Were Collected at Time 2

##### Self-Report of Offending (Huizinga et al., 1991)

The self-report of offending included 21 items from an original 23-item scale assessing behaviors such as assault, theft, and fraud. Participants reported which of these they had committed since completing the first questionnaire (approximately 6 months earlier). The questionnaire asked, “Since our last survey, have you . . .” and listed each action (e.g., “Stolen something from a store (shoplifted)?”), and participants circled “No” or “Yes.” The scale has good psychometric properties (Knight et al., 2004) including associations with theoretically linked constructs, such as arrests, peer delinquency, and impulsivity. We removed two sex-related items due to the age of the participants. For each participant, we counted how many different types of offenses had been committed.

##### Beliefs Supportive of Aggression

This is also called the *Attitude Toward Violence* scale (Houston Demonstration Project, 1993) as adapted previously (Bosworth & Espelage, 1995, as cited in Dahlberg et al., 2005). Six items assessed the belief that violence is justified in a variety of social situations, for example, “If I walk away from a fight, I’d be a coward (‘chicken’)”; α = .73 in this sample. Some validity support is provided by evidence that these items differentiate youth who bully or engage in dating violence (Bosworth et al., 1999; Smith-Darden et al., 2017). For each item, possible responses ranged from 0 “strongly disagree” to 4 “strongly agree.”

##### Little’s Pure Overt and Relational Aggression Scales (Little et al., 2003)

The pure overt scale includes physical and other types of aggression (e.g., “I’m the type of person who hits, kicks, or punches others,” α = .88). The pure relational scale includes items such as “I’m the kind of person who tells my friends to stop liking someone” (α = .83). For each item, possible responses ranged from 0 “not at all true” to 3 “very much true.” Some evidence for validity comes from correlations with self-rated and peer-rated hostility and peer-rated antisocial behavior (Little et al., 2003).

##### Gang Attitudes Subscales (Winfree et al., 1994) as adapted by Esbensen (2003)

We assessed attitudes toward gangs because gangs facilitate violence (Thornberry et al., 2002; Winfree et al., 1994) and because our community coalition was focused on gang issues. Four items assessed *gang approval* (a = .83 in our sample) by asking participants about their approval of gang-related behavior (e.g., “doing whatever the gang leaders tell you to do”). For each item, possible responses ranged from 0 “strongly disapprove” to 4 “strongly approve.” Higher scores on these items are associated with gang involvement (Esbensen, 2003; Winfree et al., 1994). *Perceived gang rewards* (α = .85 in this sample) items asked participants whether each of seven particular events would occur if they were involved in a gang (e.g., “I would get money”). *Perceived gang consequences* (α = .86) items asked whether bad consequences would occur (e.g., “I would lose my nongang friends”). The original, shorter, 10-item rewards and consequences questionnaire was developed by Winfree et al. (1994) [later expanded by Esbensen (2003)] and was correlated with gang involvement.

##### Teacher Reported Reactive and Proactive Aggression (Dodge & Coie, 1987)

Teachers provided ratings of reactive aggression (e.g., “When this teen has been teased or threatened, he or she gets angry easily and strikes back”) and proactive aggression (e.g., “This teen threatens or bullies others to get his or her own way”); α = .92 and .93, respectively, in this sample. Each subscale had three items, and possible teacher responses ranged from 1 “never true” to 5 “almost always true.” The sample size was smaller (*n* = 266) for this measure due to some teachers or parents choosing not to participate in this part of the study. Teacher ratings on these scales have been correlated with researcher-observed reactive and proactive aggression (Dodge & Coie, 1987).

#### Three Indicators of At-Risk Status Were Gathered at Time 1

##### Impulsivity

Impulsivity was assessed using the seven highest loading items from the Eysenck and Eysenck (1978) measure of impulsiveness (e.g., “I often get into a jam because I do things without thinking”). Some support for validity comes from the fact that scores on the overall scale are elevated among conduct-disordered youth (Daderman, 1999; White et al., 1994). The scale was shortened because adequate internal consistency was anticipated with fewer items (α = .83 in this sample) and because the classroom time available for the survey was limited. For each item, possible responses ranged from 0 “not at all true” to 3 “very much true.”

##### Peer Delinquency

We assessed peer delinquency with eight items asking how many of one’s friends (none, few, some, or most) had engaged in particular acts (e.g., “Used a weapon or force to get money. . .,” “stole something worth more than $100”; α = .86 in this sample). For each item, possible responses included “none,” “few,” “some,” and “most..” The items were adapted from the Rochester Youth Development Study (Thornberry et al., 1994). The original items were associated with delinquency (Thornberry et al., 2002). One item had a weaker relationship with the other items and assessed a behavior not widely perceived as lawbreaking (“Skipped classes without an excuse”), so we replaced it with a gang-related item (“How many of your friends are in a gang?”); alpha rose from .75 to .82.

##### School Failure

We assessed school failure with a single item asking, “How often do you get failing grades?” The response options were “never,” “sometimes,” or “a lot.”

### Procedures

If a parent actively consented and the youth was in class on the day of the study, the youth was provided with an assent form and a chance to participate. Youth were provided with pizza and were entered into a raffle to win an electronic item. Questionnaires were read aloud in class, and youth recorded their answers on their own copy. When consent or assent was not provided, no demographic or other information was gathered. Approximately 6 months later, a second session was scheduled. Teachers were also provided with questionnaires for the youth if parents consented. Approximately, 9% (*n* = 34) of participants in the Wave 1 survey were not present at Wave 2. The research was approved by a university ethics board.

### Analyses

First, to assess the predictive power of the indicators of optimal functioning, partial correlations were calculated between the predictors at Time 1 and the outcome variables at Time 2 (approximately 6 months later)—these partial correlations controlled for gender and age. Participants were included in any analysis for which they had sufficient data.

Second, regression analyses were conducted to assess whether optimal functioning offered predictive power beyond that provided by the risk variables. The predictors were entered in this order: demographics (gender and age), risk factors (peer delinquency, school failure, and impulsivity), and indicators of optimal functioning. Because of the many similar relations shown in Table 1, and to reduce the number of analyses, subsequent analyses examined fewer outcomes. The regression outcomes included (a) self-reported offending; (b) a composite variable computed by taking the first unrotated principal component of beliefs supportive of aggression, Little’s Aggression scales, gang approval, and perceived gang rewards; and (c) teacher reports of proactive and reactive aggression. The self-reports of offenses were kept separate from other variables because some practitioners may have a particular interest in predicting this variable. The combined self-report outcomes related to violence and aggression had similar results in Table 1, and all had loadings more than .70 on the first unrotated principal component of these variables (.77 for aggressive beliefs, .79 overt aggression, .71 reactive aggression, .73 positive gang attitudes, and .71 gang approval). Principal component analysis is primarily a data reduction method, so directly meets the goal of reducing the number of variables. The high loadings suggest that separate analyses for these would have been somewhat redundant. Third, moderation analyses were conducted to assess whether the genders differed in terms of relations between optimal functioning and outcomes, although we had no hypotheses regarding gender.

**Table 1.** Partial Correlations Between Optimal Functioning (Time 1) and Outcomes (Time 2).

	Time 2	Violent beliefs	Overt aggression	Relational aggression	Gang attitudes			Ratings by teacher	
	Offenses				Gang reward	Gang harm	Gang approval	Reactive aggression	Proactive aggression
Time 1									
Authenticity									
Living	−.17**	−.36***	−.23***	−.32***	−.21***	.31***	−.30***	−.20***	−.20***
Resisting	.08	.18	.17**	.21	.15**	−.12*	.16**	−.08	−.01
Positive regard									
Generalized trust									
Trustworthy	−.08	−.08	−.10	−.11*	−.12*	−.04	−.06	−.13*	−.04
Helpful	−.12*	−.16**	−.15**	−.20***	−.12*	−.02	.00	−.04	−.01
Fair	−.15**	−.23***	−.19***	−.25***	−.24***	.06	−.16**	−.08	−.09
Forgiveness	−.23***	−.41***	−.33***	−.37***	−.24***	.07	−.15***	−.16*	−.06
Gratitude	−.26***	−.34***	−.27***	−.33***	−.26***	.29***	−.24***	−.17**	−.06

*Note*. Gender and age were controlled for in these partial correlations.

* *p* ≤ .05. **p ≤ .01. ***p ≤ .001.

Fourth, regression analyses were used to assess whether increased risk (indicated by school failure, delinquent peers, and impulsivity) weakened relations between optimal functioning and outcomes. We would have had 21 interaction terms if we included all seven predictors and the three risk factors. Combined with four outcomes, this would have produced 84 hypothesis tests. This could increase the likelihood of chance results. Therefore, for this analysis, we used only one optimal functioning variable (the first unrotated principal component of the optimal functioning indicators that emerged as predictors in the partial correlations) and one risk variable (the first unrotated principal component of school failure, delinquent peers, and impulsivity) and their interaction term. The variables within each component shared some variance as shown by the following: Each predictor (gratitude, forgiveness, authentic living, and the three trust questions) had a loading more than .4 on the predictor principal component. Each risk indicator (impulsivity, delinquent peers, and school failure) had a loading more than .55 on the risk component. This use of principal components has potential to increase statistical power by reducing error score variance and reduce the likelihood of erroneous results by reducing the number of analyses while still achieving the goal of assessing whether optimal functioning had less predictive power for youth with elevated risk.

## Results

Table 1 shows the partial correlations between humanistic optimal functioning and outcomes. The results suggest that authentic living and positive regard (gratitude, forgiveness, and some elements of generalized trust) predict low levels of several violence-related variables 6 months later. The weakest findings were for the teacher reports of outcomes. The findings for one element of authenticity, resisting influence, were the opposite of expectations: resisting influence predicted beliefs supportive of violence and positive attitudes toward gangs.

The regression results in Table 2 suggest that indicators of optimal functioning can sometimes provide incremental predictive validity beyond that accounted for by the risk factors included here. In particular, the indicators of optimal functioning offered incremental validity for predicting the composite of violence-related self-report variables and for predicting teacher reports of reactive aggression. The results for individual optimal functioning variables are shown in Table 3. Those are the same regressions as in Table 2, but Table 2 shows overall results for each block of variables rather than results for each optimal functioning variable within the optimal functioning block.

**Table 2.** Time 1 Variable Blocks Predicting Time 2 Outcomes (Testing for Optimal Functioning Providing Prediction Beyond That Provided by Risk Factors).

Time 2	Time 1	Δ*R*^2^	*F*	*df*	*p* value
Outcomes predicted	Predictor blocks				
Self-reported offenses	Demographics	.009	1.897	2/424	.151
	Risk factors***	.166	28.269	3/421	<.001
	Optimal function	.023	1.697	7/414	.108
Violence self-report composite	Demographics	.011	2.466	2/453	.086
	Risk factors***	.202	38.466	3/450	<.001
	Optimal function***	.120	11.358	7/443	<.001
Teacher report of reactive aggression	Demographics***	.035	7.589	2/421	.001
	Risk factors*	.018	2.647	3/418	.049
	Optimal function*	.035	2.269	7/411	.028
Teacher report of proactive aggression	Demographics***	.034	7.366	2/421	.001
	Risk factors	.012	1.792	3/418	.148
	Optimal function	.027	1.709	7/411	.105

*Note*. Blocks of predictors were entered into the regressions in the following order: Demographics, risk factors, and then optimal functioning.

* *p* ≤ .05. ***p ≤ .001.

**Table 3.** Optimal Functioning Variable Results for Time 1 Predicting Time 2 Outcomes.

Time 2 outcome	Time 1 predictor	β	*t*	*p* value
Self-reported offenses	Block not significant			
Violence self-report composite score	Forgiveness**	−.134	−2.876	.004
	Gratitude**	−.122	−2.643	.009
	Trust: Trustworthy	−.036	−.885	.376
	Trust: Helpful	.030	.729	.466
	Trust: Fair*	−.099	−2.386	.017
	Authentic resisting*	.089	2.142	.033
	Authentic living***	−.179	−4.080	<.001
Teacher report of reactive aggression	Forgiveness	−.021	−.368	.713
	Gratitude	−.039	−.690	.490
	Trust: Trustworthy	−.074	−1.481	.139
	Trust: Helpful	.034	.677	.498
	Trust: Fair	−.012	−.241	.810
	Authentic resisting*	−.104	−2.052	.041
	Authentic living*	−.109	−2.038	.042
Teacher report of proactive aggression	Block not significant			

*Note*. Table 2 shows results for blocks of variables. Table 3 relies on the same regressions, but shows results for individual variables in the optimal functioning block. No beta weight is shown for a term when the addition of the optimal functioning block of variables did not produce a significant change in *R*^2^.

* *p* ≤ .05. **p ≤ .01. ***p ≤ .001.

Gender did not moderate the optimal functioning predictions except for teacher reports of proactive aggression, which was the one variable least predicted by optimal functioning. The gratitude interaction had a positive beta weight (β = .74, *p* = .004), meaning that the gratitude effect predicted more strongly for females. In contrast, authentic living had a negative beta weight (i.e., β = –.70; in the direction of authentic living predicting more strongly for males). In sum, gender interactions were the exception rather than the tendency.

As shown in Table 4, the interaction between risk level (school failure, delinquent peers, and impulsivity) and optimal functioning was significant. In particular, optimal functioning predicted more strongly for youth at otherwise greater risk of perpetrating violence. For example, in predicting offending, the beta weight on optimal functioning is –.115 when risk is at the mean, but –.395 when risk is elevated 1 *SD*. This suggests that at-risk youth may show an especially strong negative relation between humanistic optimal functioning and offending. Similarly, in predicting the self-report of violence-related variables, the beta for optimal functioning is –.346 when risk is at the mean but becomes –.494 when risk is 1 *SD* above the mean. This suggests that at-risk youth may show an especially strong negative relation between humanistic optimal functioning and violence-related outcomes.

**Table 4.** Regressions Assessing Interaction Between Optimal Functioning and Risk Level When Predicting Outcome.

Predicted Variables and Predictors	Results for blocks of predictors				Betas for individual variables			
	Δ*R*^2^	*F*	*df*	*p* value	Variables	β	*t*	*p* value
Predicted: Self-reported offenses								
Demographics	.009	2.026	2/453	.133				
Opt. Funct. and Risk***	.176	48.554	2/451	<.001***	Optimal. Funct.**	−.115	−2.619	.009
					Risk***	.279	6.180	<.001
Interaction of Opt. Funct. and Risk***	.071	43.138	1/450	<.001***	Opt. × Risk***	−.280	−6.568	<.001
Predicted: Violence self-report composite score								
Demographics	.011	2.466	2/453	.086				
Opt. Funct. and Risk***	.271	84.881	2/451	<.001	Optimal. Funct.***	−.346	−8.107	<.001
					Risk***	.234	5.364	<.001
Interaction of Opt. Funct. and Risk***	.020	12.843	1/450	<.001	Opt. × Risk***	−.148	−3.584	<.001
Predicted: Teacher report of proactive aggression: Interaction not significant								
Predicted: Teacher report of reactive aggression: Interaction not significant								

*Note*. Beta values reflect the regression equation after the last statistically significant block of variables had been added. Thus, no beta weight is shown for a term when the addition of that block did not produce a significant change in *R*^2^.

** *p* ≤ .01. ***p ≤ .001.

A reviewer offered the insightful comment that authenticity may operate differently amid collectivism. As a first response, we examined the correlation between collectivism and the authenticity measures but found no statistically significant result. Testing interactions with collectivism seemed a reasonable next step, but we were hesitant to test many cultural interactions because this path could result in many hypothesis tests (and the related problems of increasing family-wise error). Thus, we focused on conducting four regressions testing the interaction between an indicator of collectivism and authenticity in predicting the outcomes. In particular, a dichotomous collectivism variable was created that identified individuals born in collectivistic countries. Significant interactions suggested that the authentic living variable may have less predictive power among the collectivistic participants for prediction of total offenses, the composite self-report violence variable, and for teacher reports of proactive aggression. The results suggest this is an avenue worthy of further exploration, but we are hesitant to make strong conclusions, in part because this indicator of collectivism was confounded with other variables such as being a cultural minority and possibly other cultural variables such as vertical cultural orientation (Sivadas et al., 2008; Triandis, 1996). It is worth considering the possibility that in collectivistic cultures, affirming the value of authenticity may have a different meaning that lacks a protective element. We are hesitant, however, to make strong conclusions from these culture-related interactions although they do raise interesting possibilities.

## Discussion

The results of these analyses suggest that humanistic optimal functioning has relevance to youth violence. Authentic living and positive regard for others (generalized trust, gratitude, and forgiveness) predicted low levels of violence-related variables approximately 6 months later. The indicators of optimal functioning offered incremental predictive power beyond that provided by the risk factors. Interactions suggest that the predictive power of humanistic optimal functioning may be greatest among youth at heightened levels of risk.

The results may seem self-evident to humanistic psychologists, but violence researchers may seldom draw from humanistic psychology for predictive variables (Coupland & Olver, 2020). Thus, there is value in providing empirical evidence for the relevance of humanistic psychology. Humanistic optimal functioning may deserve inclusion in assessments of violence potential and, if these effects are causal, within prevention and intervention efforts. Indicators of optimal functioning may be especially relevant for youth scoring high on risk indicators.

Authentic living, one of the indicators of optimal functioning studied here, predicted low violence-related outcomes. Authenticity has long occupied a central role in humanistic psychology (Rogers, 1957; Tohme & Joseph, 2020). Authenticity is an interesting variable because it has been valued among philosophers (Sartre, 1975) and within popular media, including even children’s entertainment in the form of exhortations to follow one’s passions. Moreover, authenticity is associated with personal well-being (Sutton, 2020). It was surprising that the authenticity subscale of resisting influence predicted higher rates of violence-related outcomes in the cross-lag partial-correlations. Perhaps the resisting influence measure, in this case, particularly captured readiness to reject messages from authority figures, something commonly seen in adolescents. This type of resistance among adolescents could possibly tend to promote or at least predict violence-related acts or dispositions.

On the suggestion of a reviewer, we tested interactions between collectivism and authenticity. Authenticity may have a different meaning in a collectivistic context. The results suggested that the authentic living variable may have less predictive power among collectivistic youth. This finding may be worth exploring in future research. The measure of collectivism we had available was far from ideal, however, so although the finding is intriguing, we suggest treating this collectivism result as unclear evidence for now.

Positive regard for others, another indicator of optimal functioning studied here, was assessed with indicators of generalized trust, forgiveness, and gratitude. Forgiveness requires letting go of resentment and having a more positive view of others who have brought harm. Gratitude requires a willingness to consider the positive benefit that others have brought to one’s life. These both require more positive regard rather than their opposite.

Generalized trust predicted lower levels of violence-related outcomes here, and it has broader relevance. Trust is also associated with psychological well-being (Oishi et al., 2011). When discussing generalized trust, it is important to represent the construct accurately. We used the word “trust” because that is the label used by other researchers (Mewes et al., 2021), but in laypersons’ usage, trust denotes making oneself vulnerable to harm from those on whom you rely. Youth, maybe especially youth who live in risky environments, need to be careful regarding in whom they place their trust. Shusako Endo (2008) wrote engagingly about the wonderful fool who trusted broadly, brought positive change to others, but suffered much harm. This study, however, and the prior studies, did not assess whether participants made themselves vulnerable to others but instead assessed the belief that most people are trustworthy. A person who scores high may still be careful to avoid risk. The term “faith in humanity” may communicate the idea better, although it encompasses more than generalized trust (Tweed et al., 2021). Concerningly, generalized trust has declined across recent decades in American samples (Mewes et al., 2021).

Forgiveness and gratitude also predicted outcomes in this study. For forgiveness, one could speculate that the effect occurs because forgiveness displaces vengeful attitudes (Berry et al., 2005) that can motivate violence (Kennedy, 2011). For gratitude, some work suggests it contributes to prosocial intentions (Tsang, 2006) and reduces antisocial behavior more broadly (Bono et al., 2019). The results here are consistent with those findings.

Tools assessing protective factors for violence can have great value (de Vogel et al., 2011). Trait forgiveness, faith in humanity (Tweed et al., 2021), authentic living, and gratitude do not tend to be included in these devices (e.g., Coupland & Olver, 2020). Perhaps that deserves reconsideration.

Some limitations deserve mention. The relevance of a school sample to clinical populations could be debated. Nonetheless, the negative relation between indicators of optimal functioning and violence-related outcomes was strongest for youth at risk, suggesting relevance to more troubled samples. In addition, offense data and much of the violence data were self-reported. In addition, follow-up beyond 6 months would have value. Furthermore, the mechanism of the authentic living effect deserves further research. Authentic living may restrain violence. Alternatively, one could consider that society permits only some people to be authentic (those with conforming values) while pressuring nonconformers to hide their true self and beliefs. It could be that people who match societal norms are given permission to be themselves and the resulting positive social experience may restrain violence. Another limitation results from the fact that the generalized trust measure used here had problems of internal consistency, so the items were kept separate as has been done previously for the same reason (Oishi et al., 2011). The measure has a long history (Rosenberg, 1956), has evidence for convergent validity from expected correlations with other variables (Brehm & Rahn, 1997; Rahn & Transue, 1998; Stavrova & Ehlebracht, 2016; Twenge et al., 2014), and has generated large effect sizes (Elgar & Aitken, 2011). Perhaps it has worked sufficiently well in the past in spite of low internal consistency because the items tap distinct domains within generalized trust; however, other measures of similar constructs have been developed (Kaufman et al., 2019; Stavrova & Ehlebracht, 2016). Because of problems with the three-item trust measure, future studies may benefit from using one of the more recently developed measures.

One caution deserves special mention. These results could easily be misinterpreted, most likely by people who are not humanistic theorists, to suggest that indicators of optimal functioning are largely fixed characteristics of individuals. To the extent that optimal functioning is unchangeable, then youth lacking these indicators could be seen by some as being doomed to a proclivity for violence. In contrast, evidence suggests that many widely known predictors of violence are dynamic (Douglas & Skeem, 2005). Thus, the potential for violence can change over time within individuals, possibly depending partly on their current environment. Thus, these indicators of optimal functioning could be changeable rather than fixed traits.

## Conclusion

Several indicators of optimal humanistic functioning predicted lower scores on measures potentially related to violence. The indicators included authentic living and positive regard toward others (generalized trust, gratitude, and forgiveness). The optimal functioning measures sometimes provided incremental prediction beyond that offered by the risk factors included here. The predictive power of optimal functioning tended to be greatest among youth at heightened levels of risk (i.e., youth who were impulsive, had trouble in school, and had delinquent peers). The predictive power of optimal functioning indicators has implications for the assessment and possibly even reduction of risk for violence.

To apply these results, assessment experts could more often include humanistic variables in their assessments. These analyses support that approach. Many youth, however, live in conditions that inhibit flourishing. To promote optimal functioning, community psychologists could seek to improve societal conditions, and therapeutic experts could provide interventions for individuals. Admittedly, however, the analyses here provide evidence of longitudinal predictive power and a possibility of causality but cannot provide proof of the causal path toward violence reduction. Thus, directly testing the impact of humanistic interventions on violence will continue to have value, especially in light of the fact that even interventions that seem obviously valuable, sometimes fail to produce their expected effect (Gelfand et al., 2022) and in light of the potential power of efficacy data for persuading others.

One significant concern exists. A focus on characteristics of persons potentially prone to violence could evoke a tendency to blame the individuals for their risk level or for not showing optimal functioning. In contrast, a humanistic perspective suggests humans are malleable and can grow in response to healthy environments. The humanistic psychology perspective suggests that the path forward involves not blaming and marginalizing but instead building conditions facilitating positive regard, empathy, and compassion toward those at increased risk of acting out in violence (Rogers, 1957).
